# A CARS-SPA-GA Feature Wavelength Selection Method Based on Hyperspectral Imaging with Potato Leaf Disease Classification

**DOI:** 10.3390/s24206566

**Published:** 2024-10-12

**Authors:** Xue Li, Xueliang Fu, Honghui Li

**Affiliations:** College of Computer and Information Engineering, Inner Mongolia Agricultural University, Hohhot 010011, China; nikolauxmas@163.com (X.L.); lihh@imau.edu.cn (H.L.)

**Keywords:** hyperspectral imaging technique, feature selection, machine learning, potato early blight, ladybug beetle

## Abstract

Early blight and ladybug beetle infestation are important factors threatening potato yields. The current research on disease classification using the spectral differences between the healthy and disease-stressed leaves of plants has achieved good progress in a variety of crops, but less research has been conducted on early blight in potato. This paper proposes a CARS-SPA-GA feature selection method. First, the raw spectral data of potato leaves in the visible/near-infrared light region were preprocessed. Then, the feature wavelengths were selected via competitive adaptive reweighted sampling (CARS) and the successive projection algorithm (SPA), respectively. Then, the two sets of wavelengths were reorganized and duplicates were removed, and secondary feature selection was conducted with genetic algorithm (GA). Finally, the feature wavelengths were fed into different classifiers and the parameters were optimized using a real-coded genetic algorithm (RCGA). The experimental results show that the feature wavelengths selected by the CARS-SPA-GA method accounted only for 9% of the full band, and the classification accuracy of the RCGA-optimized support vector machine (SVM) classification model reached 98.366%. These results show that it is feasible to classify early blight and ladybug beetle infestation in potato using visible/near-infrared spectral data, and the CARS-SPA-GA method can substantially improve the accuracy and detection efficiency of potato pest and disease classification.

## 1. Introduction

Potato is the fourth-largest grain crop after wheat, corn and rice in China. The high and stable yield of potato is of great significance to ensure food security in China. Inner Mongolia is one of the most important production areas for potato planting. According to the “Current Trend Forecast of Major Crop Diseases and Pests in Inner Mongolia Autonomous Region in 2023” [1], released by the Agriculture and Pastoral Department of the Inner Mongolia Autonomous Region, early epidemics and ladybug pests pose a great threat to the potato yield in Inner Mongolia. Although early blight mainly occurs in the late growth stage of potato, the potato yield is also strongly affected when the disease is serious [2]. Therefore, research on the efficient and non-destructive monitoring of pest and disease stress conditions in potato is important for pest and disease control.

The traditional detection method is mainly based on subjective identification by experienced potato farmers by the naked eye, but this method is time-consuming and labor-intensive due to the wide range of potato cultivation and irregular onset times. Detection by chemical methods is more accurate, but it is time-consuming, expensive and not yet as convenient as visual inspection. Thus far, certain research progress has been made in the identification and classification of pests and diseases of various crops based on machine vision [3], and the classification model has gradually changed from machine learning to deep learning. In order to obtain higher classification accuracy, deep learning requires the input of more data and its learning depth must be continuously increased to adapt to recognition difficulties such as noise and complex backgrounds in pest and disease recognition. This will bring about longer model training times accompanied by time-consuming and labor-intensive manual labeling work. Deep learning feature extraction strategies do not rely on feature engineering, but the extracted data cannot be analyzed for features and the algorithms are less interpretable. Machine learning, on the other hand, can better balance the performance and complexity of the model, is more suitable for processing small samples and high-dimensional hyperspectral data and can be combined with feature engineering to realize the needs of spectral data analysis [4].

Early blight is a fungal disease that starts mainly at the flowering and fruiting stage of the crop [5] and mainly affects a wide range of Lycopersicon crops, such as potato, tomato, cucumber, eggplant and tobacco [6]. There are fewer studies on early blight in potato based on hyperspectral techniques. Xu Mingzhu et al. [7] utilized SPA and the load factor method for feature wavelength extraction with a BP model and SVM model, and the final selected features only accounted for 1.47% of the original bands. Li Xinting et al. [8] successfully classified the leaves of potato under early blight at different disease stages using convolutional neural networks combined with different preprocessing and feature extraction methods. However, these studies only classified healthy and early blight leaves, with a single classification objective and no consideration of the ability of the classification model to distinguish spots similar to those of early blight.

Feature wavelength selection is a critical step in spectral analysis that aims to identify the wavelengths that best represent the sample properties from a large number of candidate wavelengths. This process is essential to improve the accuracy of the analysis, reduce the complexity of the model and enhance the generalization ability of the model. To date, a number of studies have combined hyperspectral techniques with feature selection and applied them to crop disease research in order to identify the key bands for disease classification and to improve the efficiency and reliability of disease classification models. Liu Li et al. [9], based on the hyperspectral data of Dangshan Crisp Pear leaves, selected wavelengths with five commonly used feature selection algorithms, such as principal component analysis and random jumping frog, and input them into different types of models. The optimal model’s classification accuracy reached 93.25%, and it was able to accurately identify the healthy leaves and differentiate between anthracnose and black spot disease, which have similar onset symptoms. Furlanetto et al. [10] developed a linear discriminant analysis (LDA) model to grade the severity of potassium-deficient leaves in soybeans by downscaling the data using the “Stepwise” program in the SAS software v9.4. To cope with different spectral data, some researchers have concatenated feature selection methods to further reduce the data dimensionality and improve the model performance. Alternatively, some concatenate different feature selection methods to comprehensively leverage their advantages. Wang Xinye et al. [11] proposed a second principal component method to select the characteristic wavelengths of potato leaves for the hyperspectral analysis of late blight. This method combines the weight coefficient curve of principal components after principal component analysis (PCA), takes several wavelengths with large weights as the input of the second PCA and selects obvious disease images from the resulting PCA images to participate in disease identification. The image disease characteristics obtained by this method are obvious, and it can effectively improve the recognition accuracy. Li Yang et al. [12] proposed a hyperspectral-based characteristic wavelength extraction method to identify cucumber downy mildew and flies. In this method, the classical algorithm is used for feature extraction, and then the continuous projection algorithm is used to extract the extracted bands twice. Among them, the SVM model, established by using the bands processed by the iterative spatial shrinkage method and continuous projection algorithm, had the best effect, with accuracy of 96.69%. These studies have extracted important bands for specific diseases in particular crops, but these characteristic bands are usually not directly applicable to other diseases.

Thus far, hyperspectral-based research on the disease detection of Solanaceae crops has achieved good progress. For example, Gold et al. [13], which related physiological changes in early and late blight potato leaves to the spectrum, developed a PLS-DA model based on in situ spectroscopy. The model distinguished the two diseases with more than 80% accuracy at 2–4 days before the onset of visible symptoms. It identified leaves with different symptoms of each disease with 95% accuracy and demonstrated the importance of the short-wave infrared band in distinguishing between the two diseases. Liu Yongchang [14] classified leaves infected by tobacco mosaic virus and potato Y virus based on tobacco leaf hyperspectral data and established SVM, K nearest neighbor and random forest discrimination models, respectively. Among them, the SVM classification model had the best classification effect, with accuracy of 90.72%. Zhang et al. [15], considering the limitations of a single monitoring method, fused NIR spectral data and THz spectral data on tomato leaves to establish a BPNN model, and the identification accuracy for tomato leaf mildew disease reached 97.12%. The above studies used different types of spectral techniques to achieve higher accuracy in the identification of pests and diseases in Solanaceae crops, but the raw spectral data processed were characterized by high complexity, high dimensionality, redundant information and large data processing costs. In this paper, the healthy, early blight and ladybug beetle-infested leaves of potato were taken as research objects, and the spectral images in the visible/near-infrared region of the leaves were examined. These were used to explore efficient methods for the identification of potato pests and diseases and to select the characteristic wavelengths for potato pest and disease identification, so as to provide empirical support for the development of unmanned monitoring equipment. The research in this paper includes (1) comparing the noise reduction effects of different preprocessing methods on potato leaf hyperspectral data; (2) analyzing the visible/near-infrared spectral curves of potato leaves; (3) proposing a CARS-SPA-GA feature wavelength selection method; and (4) establishing the RCGA-SVM, RCGA-RF and RCGA-ANN potato leaf disease classification models.

## 2. Materials and Methods

This section describes in detail the acquisition of the experimental samples, the acquisition and processing of the sample hyperspectral data, the CARS-SPA-GA feature selection method, the model building, the optimization of the model’s parameters and the model evaluation method.

### 2.1. Experimental Potato Leaves

**Figure 1:**
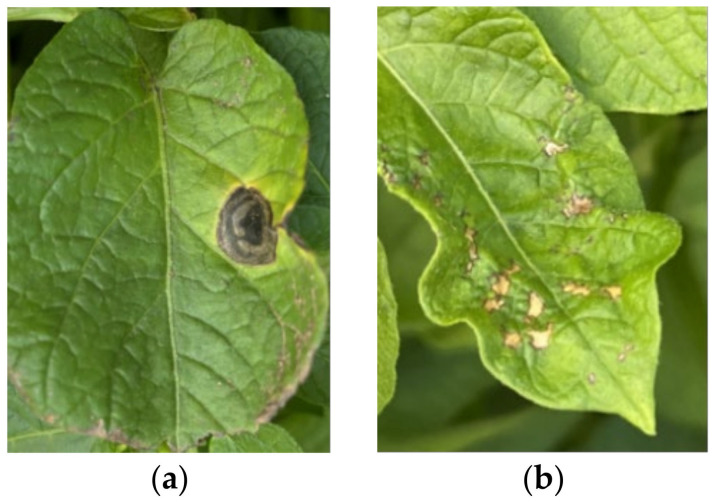
Potato leaves under disease and insect pest stress. (**a**) early blight infestation; (**b**) ladybird beetle infestation.

The experimental samples were leaves of potatoes grown in field environments in Wuchuan County, Hohhot City, Inner Mongolia Autonomous Region and Chahar Right-Wing Qian Banner, Ulanqab City, including 48 healthy leaves, 48 early blight leaves and 48 ladybug beetle-infested leaves. The collections were performed on 31 July and 6 August 2023, when the potatoes in the experimental area were in the flowering stage. All plants were infected with early blight and ladybugs in the natural state. The leaves are shown in Figure 1; Figure 1a shows the potato leaves infected with early blight, and Figure 1b shows the potato leaves infected by ladybugs.

### 2.2. Experimental Hyperspectral Imagery

**Figure 2:**
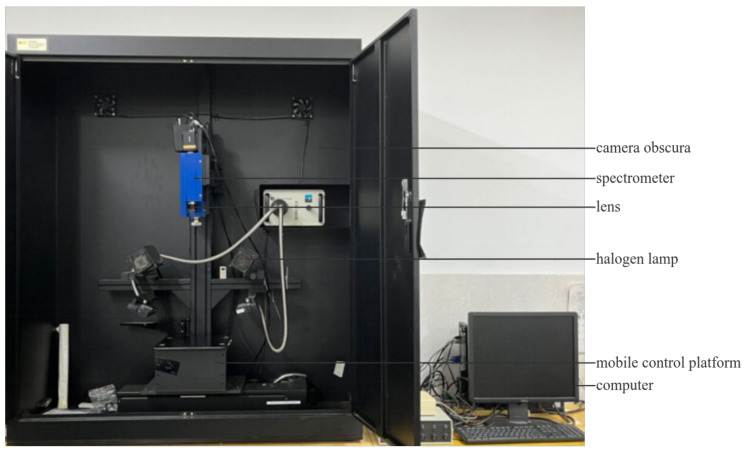
Hyperspectral imaging system.

The experiment using a ISUZU OPTICS HSI system (Zhubei City, Taiwan) yielded spectral information in the 382.2358~1004.7804 nm range with a band number of 428 and a spectral resolution of 2.8 nm. As shown in Figure 2, the experiment was carried out in a black box, and the system included a hyperspectral image spectrometer (Spectral Imaging Ltd., Oulu, Finland), a CCD camera (Imperx, Boca Raton, FL, USA), two halogen lamps (150 W) (Illumination Technologies, Atlanta, GA, USA), a DC adjustable light source (Illumination Technologies, Atlanta, GA, USA), a mobile control platform (ISUZU OPTICS, Taiwan, China), a computer, etc. Before the experiment, black-and-white correction was performed to eliminate the influence of the background, dark current and light inequality on the reflectivity. The calculation formula is as follows:

(1) $$R=\frac{I_{S}-I_{D}}{I_{W}-I_{D}},$$

R is the relative spectral reflectance of the potato leaf samples, $I_{S}$ is the original spectral intensity of the potato leaves, $I_{D}$ is the reflection spectrum intensity of the reference blackboard and $I_{W}$ is the reflection spectrum intensity of the self-made whiteboard.

### 2.3. Data Extraction of Regions of Interest

**Figure 3:**
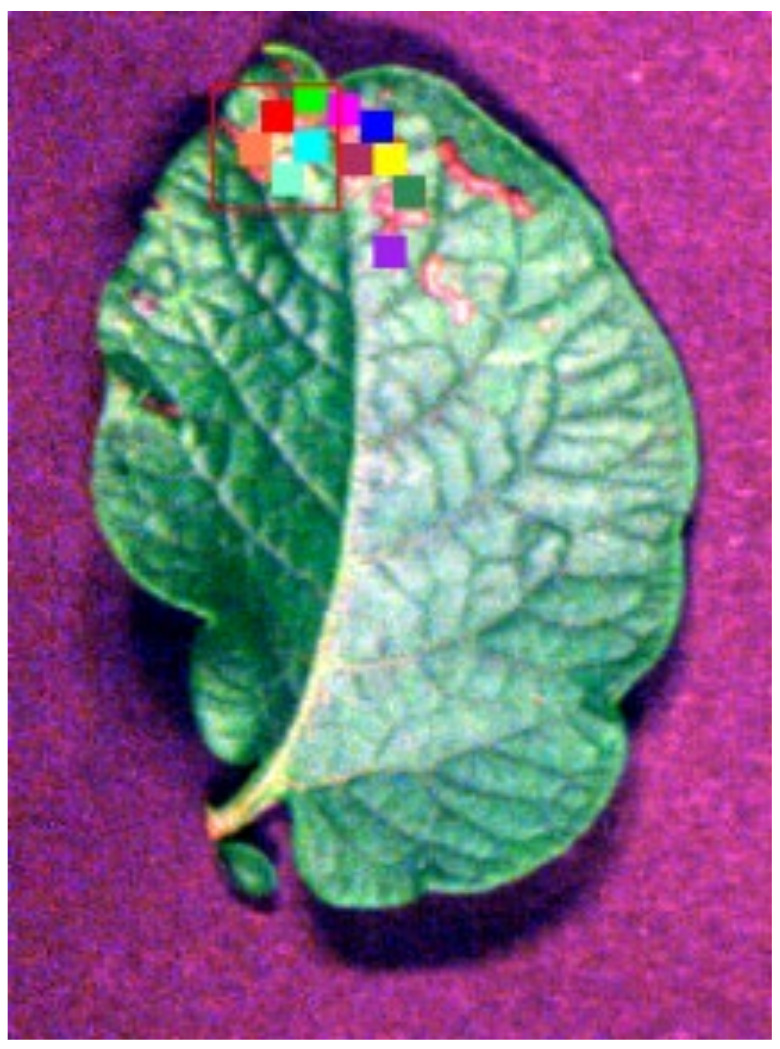
A schematic diagram of the ROI selection process.

The hyperspectral images of the leaves were opened using ENVI 5.6, and a 10 × 10 pixel area was manually selected as the region of interest (ROI). As shown in Figure 3, each colored rectangular block represents an ROI. This area, by avoiding strong reflections and darker pixel points and including representative parts, represents the smallest possible pixel area, resulting in a more adequate sample size. The average reflectance of the ROI was calculated as raw spectral data and labeled categories, where healthy leaves were labeled as 0, early blight leaves as 1 and worm-eaten leaves as 2. For the purpose of avoiding the influence of the uneven number of samples in different categories on the experiment, the samples of the three categories were censored; finally, 1017 samples were obtained, with 339 samples in each category.

### 2.4. Data Preprocessing

Since hyperspectral data are susceptible to factors such as the experimental environment and the noise of the instrument itself, in this paper, the data with obvious noise at the beginning and end of the spectrum are removed, retaining 276 bands in the range of 450.0800–850.8145 nm. Five preprocessing methods, namely the Savitzky–Golay filter (SG) [16], standard normal variable (SNV) [17], multiplicative scatter correction (MSC) [18], Min–Max scaler (MMS) [19] and wavelet transform (WT) [20], were utilized to preprocess the data with a view to finding the optimal preprocessing method that could reduce the effects of leaf surface reflections, light range variations and dark currents.

### 2.5. Feature Wavelength Selection

In this paper, we use the competitive adaptive reweighted sampling method (CARS) [21], the successive projection algorithm (SPA) [22] and genetic algorithm (GA) [23] to select features for the preprocessed full-band data, and we propose a CARS-SPA-GA feature selection method based on the experimental results.

CARS can effectively handle data redundancy and covariance. This algorithm combines the idea of importance sampling in Monte Carlo sampling with the regression coefficients of the partial least squares (PLS) model and uses the absolute value of the regression coefficients in the PLS model as the object of importance assessment [24]. First, the points with larger absolute values are taken as the new subset by ARS, and the points with smaller absolute values are eliminated; then, the PLS model is re-established based on the current subset, and the wavelength in the subset with the smallest RMSECV of the PLS model is the optimal feature wavelength after several iterations.

SPA is a forward iterative search algorithm [25] that is simple to implement, fast to compute, suitable for processing high-dimensional data and commonly used in spectral analysis research. This algorithm calculates the projection on other unselected wavelengths based on the selected initial wavelength, adds the unselected wavelength corresponding to the maximum projection to the wavelength combination, establishes the PLS model and calculates the RMSE, and the wavelength combination with the smallest RMSE is the optimal wavelength combination. Considering the advantage whereby SPA can effectively reduce the dimensionality of the data, as well as the complexity of the data itself, the range of the number of features selected for SPA was set to 20–50 in the experiment.

GA is a global search algorithm whose search process is motivated by a fitness function. When GA is applied to the feature selection problem, it yields a binary code that encodes the genes in a chromosome as either 0 or 1, so that a chromosome is a string containing 0 and 1. Where genes correspond to features, 0 means that the feature is not selected and 1 means that the feature is selected, and chromosomes correspond to combinations of features.

**Figure 4:**
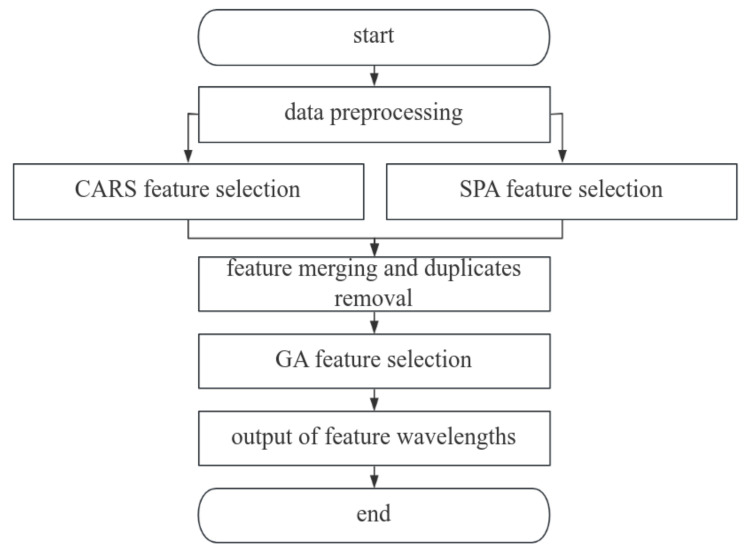
The CARS-SPA-GA feature selection flow diagram.

Feature selection is a complex, large-scale problem, and GA is prone to converge prematurely when facing such problems, thus falling into local optimal solutions. Therefore, a CARS-SPA-GA feature selection method is proposed in this paper, as shown in Figure 4. Firstly, the preprocessed data are subjected to feature selection by the CARS and SPA algorithms. Then, the obtained two sets of feature wavelengths are combined and the duplicates are removed. Finally, GA is used for secondary feature selection, and the output feature wavelength is the final preferred feature wavelength.

### 2.6. Model Building for Classification of Potato Leaf Diseases

Machine learning and deep learning models have been widely used in plant spectral data learning, including hyperspectral data on leaves, fruits, seeds, canopies, etc. The most commonly used classifiers for such data are support vector machine (SVM) [26,27], random forest (RF) [28,29], multilayer perceptron (MLP) [30], partial least squares discriminant analysis (PLS-DA) [31,32], etc. In this study, three applicable algorithms from machine learning, integrated learning and deep learning were selected for model building and were used to explore more suitable modeling algorithms for spectral data.

SVM is a kernel learning algorithm for small sample datasets as well as high-dimensional datasets. The potato leaf hyperspectral data collected in this study have 276 features that are nonlinearly correlated, so the experiment selects the radial basis kernel function (RBF kernel) for SVM, the mathematical expression of which is shown in Equation (2). It efficiently handles high-dimensional data and solves nonlinear problems by mapping samples from the original space to a high-dimensional space where they are linearly differentiable [33].

(2) $$K\left(x_{i},x_{j}\right)=exp\left(-\gamma {\left\|x_{i}-x_{j}\right\|}^{2}\right),$$

where $K\left(x_{i},x_{j}\right)$ is the value of the kernel function between two data points $x_{i}$ and $x_{j}$, γ is an adjustable parameter controlling the width of the kernel and $\left\|x_{i}-x_{j}\right\|$ is the Euclidean distance between two data points.

RF is a representative supervised learning algorithm in ensemble learning. It consists of multiple decision tree classifiers, each of which draws random samples from the original data in a put-back set during training, which increases the diversity of the model and helps to reduce overfitting. The model outputs the final result after voting on the classification results of all decision trees.

An artificial neural network (ANN) is a deep learning algorithm that simulates the neural workings of the human brain. The MLP used in this paper is an ANN with a relatively simple structure. The MLP is able to capture and learn complex patterns and nonlinear relationships in data through its multilayer structure and nonlinear activation functions, which is particularly important for classification tasks. The MLP model established in this paper consists of three layers: an input layer, hidden layer and output layer. The number of neurons in the input layer coincides with the number of bands in the input, and the output layer has three neurons, consistent with the sample categories, representing healthy, early blight and insect-stressed potato leaves.

### 2.7. Parameter Optimization of Classification Models with Real-Coded Genetic Algorithm

**Figure 5:**
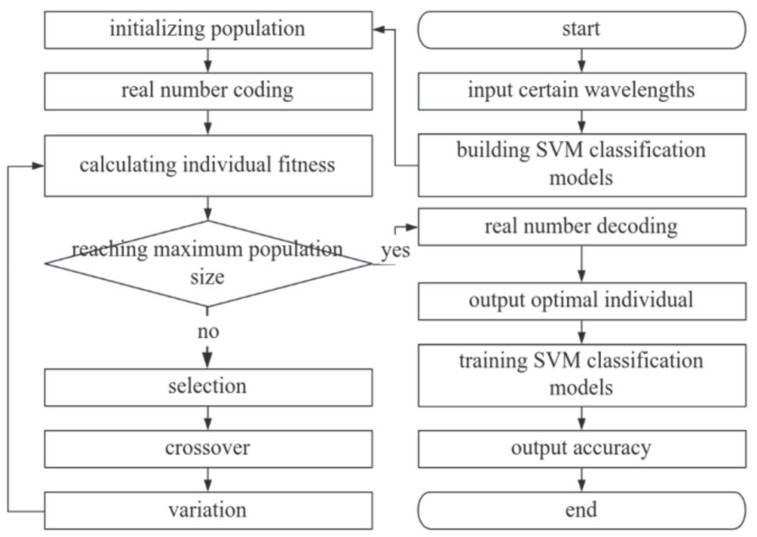
Flowchart of RCGA in optimizing the parameters of an SVM model.

SVM possesses a strong generalization ability, even for unknown data, but it is very sensitive to the parameter settings, which makes parameter optimization a key step in improving the performance of SVM models. The genetic algorithm (GA) is an intelligent global optimization algorithm that has been widely used and is quite effective. For cases in which the feasible solution of the optimization problem is real, the commonly used binary coding approach is obviously not applicable, so the real-coded genetic algorithm (RCGA) [34] is utilized to optimize the model parameters. The parameters include the penalty coefficient C and kernel function coefficient gamma of SVM; the number of classifiers and maximum depth of RF; and the learning rate and the number of hidden unit layers plus the number of neurons from the MLP. Figure 5 shows the flowchart for the optimization of the parameters with the RCGA, using SVM as an example.

The specific steps of the RCGA in optimizing the parameters of the SVM classification model are as follows.

1. Potato leaf hyperspectral data and tag data are fed into the SVM classifier.
2. The parameters of the real-coded genetic algorithm are initialized, and the initial populations containing the parameters C and gamma are generated and encoded.
3. The classification accuracy is calculated as the individual fitness based on the fitness function.
4. It is determined whether the current population number has reached the threshold value; if it is not the maximum value, proceed to step 5; if it is the maximum value, proceed to step 8.
5. Individuals are selected by the roulette method.
6. Crossing between individuals is performed according to the arithmetic crossing mode, and the population is updated. The arithmetic crossing formula is as follows:
  (3) $$X_{ij}^{\prime }=\alpha X_{ij}+(1-\alpha )X_{(i+1)j},$$
  (4) $$X_{(i+1)j}^{\prime }=(1-\alpha )X_{ij}+\alpha X_{(i+1)j},$$
  where $X_{ij}$ is the j-site gene of the current individual i, $X_{(i+1)j}$ is the j-site gene of the neighboring individual (i + 1), $X_{ij}^{\prime }$ is the j-site gene of the new individual i, $X_{(i+1)j}^{\prime }$ is the j-site gene of the new individual (i + 1) and α is the percentage of the non-crossover portion of the individual’s genes.
7. The mutation of individual genes is performed using a single point mutation, which is calculated as follows.
  When θ is greater than 0.5,
  (5) $$X_{ij}^{\prime }=X_{ij}-(X_{ij}-X_{min})(1-r^{\frac{T-t}{T}});$$
  when θ is less than or equal to 0.5,
  (6) $$X_{ij}^{\prime }=X_{ij}+(X_{max}-X_{ij})(1-r^{\frac{T-t}{T}}),$$
  where $X_{ij}$ is the current j-site gene of individual i; $X_{ij}^{\prime }$ is the j-site gene of individual i after the mutation; $X_{max}$ and $X_{min}$ are the upper and lower limits of the j-site gene, respectively; T is the maximum number of evolutions; t is the current number of evolutions; and θ and r are random numbers with values in the range $\left[0,1\right]$.
  Then, steps 3 and 4 are repeated.
8. The current optimal individual is decoded and output as the parameters C and gamma.

### 2.8. Model Evaluation

The models are evaluated using the overall accuracy (OA), confusion matrix and kappa coefficient. The OA is a common evaluation metric for the assessment of the performance of classifiers and is calculated as follows:

(7) $$OA=\frac{T_{0}+T_{1}+T_{2}}{T_{0}+N_{0}+T_{1}+N_{1}+T_{2}+N_{2}},$$

where $T_{0}$, $T_{1}$ and $T_{2}$ are the numbers of samples correctly categorized in each category, and $N_{0}$, $N_{1}$ and $N_{2}$ are the numbers of samples incorrectly categorized in each category. A confusion matrix is a visualization tool that presents the model classification results and helps us to understand the strengths and weaknesses of the model in classifying samples in each category. The kappa coefficient is a measure based on the confusion matrix, and, unlike the OA, which only takes into account correctly categorized samples, it also takes into account the effect of incorrectly categorized samples, and it is calculated as follows:

(8) $$kappa=\frac{OA-p_{e}}{1-p_{e}},$$

where $p_{e}$ is calculated as follows:

(9) $$p_{e}=\frac{a_{1}\times b_{1}+a_{2}\times b_{2}+a_{3}\times b_{3}}{n\times n},$$

where $a_{1}$, $a_{2}$ and $a_{3}$ represent the true numbers of samples in each category; $b_{1}$, $b_{2}$ and $b_{3}$ represent the predicted numbers of samples in each category; and n represents the total number of samples.

## 3. Results and Discussion

### 3.1. Spectral Analysis

**Figure 6:**
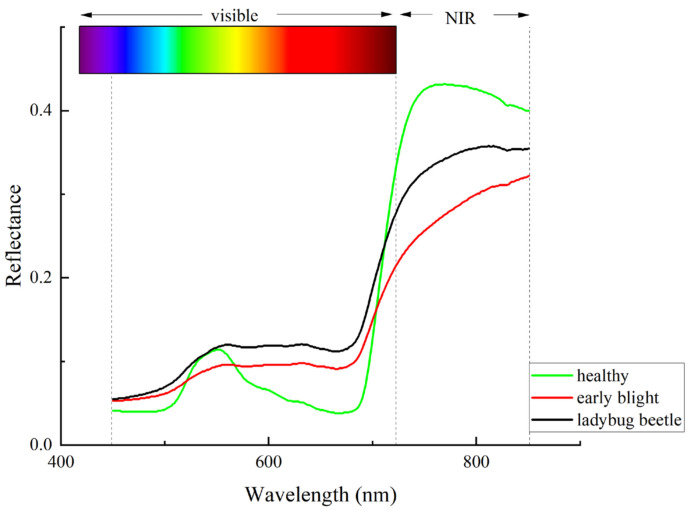
Comparison of spectral curves of three types of potato leaves.

The spectral property of plant leaves is determined by their internal structure, chlorophyll, water, etc. [35]. The hyperspectral data of the three types of potato leaves were averaged over the full wavelength band and then visualized using the Origin software 2021 and divided into two regions, namely the visible and near-infrared regions. The average spectral curves of the three types of leaves are shown in Figure 6. Among them, due to the strong absorption of blue and red light by chlorophyll and the weak absorption of green light, the healthy potato leaves formed obvious absorption peaks near the blue light absorption band at 480 nm and the red light absorption band at 680 nm, as well as obvious reflection peaks near the green light region at 550 nm, which is in agreement with other scholars’ studies on the spectral characterization of potatoes [36]. The spectral curves of the three types of potato leaves are roughly similar: there is an obvious absorption peak at 680 nm, and the reflectance rises sharply at 680~750 nm due to the “red edge” effect, showing that the reflectance of the near-infrared region on the right side of the “red edge” is significantly higher than that of the visible region on the left side of the “red edge”. The reflectance of healthy leaves is lower than that of diseased and insect-infested leaves in the range of 450~580 nm, and there is an obvious reflectance peak at 550 nm in the curves of the healthy leaves, while those of the diseased and insect-infested leaves are more flat, probably due to the damage to these leaves and thus the decomposition of chlorophyll into carotenoids and lutein, which weakens the absorption of blue–violet light and has high reflectance. Chlorophyll is the main absorbing substance of red light. In the red light range of 580~720 nm, diseased leaves are damaged and the chlorophyll content decreases, which leads to a reduction in the absorption of red light by the leaves, resulting in higher reflectance than healthy leaves. In the red and near-infrared region to the right of 720 nm, the reflectance of diseased leaves is lower than that of healthy leaves, probably due to the damage to the cellular structure of the diseased leaves, which has an effect on the reflectance.

### 3.2. Model Parameter Settings

The experiment was based on the Python 3.7 programming environment, and the dataset was divided into a training set and test set according to a 7:3 ratio. The settings of the following parameters were decided based on research experience using the trial and error method and validated by the cross-validation method, using the classification accuracy as a decision criterion. In the wavelength selection algorithm, the number of GA population evolutions is 50, the population size is 90, the crossover probability is 0.8 and the mutation probability is 0.5. The maximum number of CARS iterations is 100, the maximum number of principal components is 20 and the number of cross-validations is 10. The maximum and minimum number of end elements of SPA are 20 and 5, respectively. In the RCGA parameter optimization algorithm, the maximum number of populations is 100, the number of population evolutions is 20, the crossover and variance rates are both 0.8 and the proportion of non-crossover parts is 0.5.

### 3.3. Data Preprocessing

**Figure 7:**
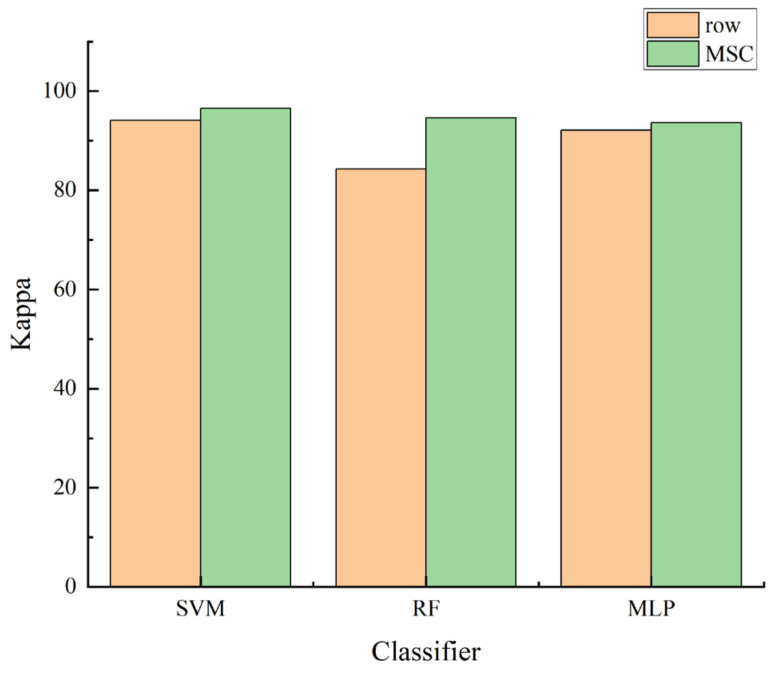
Comparison of effectiveness enhancements of MSC preprocessing methods on different classifiers.

Appropriate preprocessing methods can reduce the interference of noise in the data and improve the classification accuracy. As can be concluded from the comparative experimental results in Table 1, the MSC preprocessing methods used in this experiment are able to eliminate the obvious noise in the spectrum. As can be seen from the visualization results in Figure 7, after preprocessing by the MSC method, the kappa of the data is substantially improved for the SVM and RF classifiers, and there is a small decrease for the MLP classifier. Hence, the subsequent experiments continued to use the MSC method to preprocess the data.

### 3.4. Feature Selection

#### 3.4.1. Feature Wavelength Selection Based on CARS, SPA and GA

**Figure 8:**
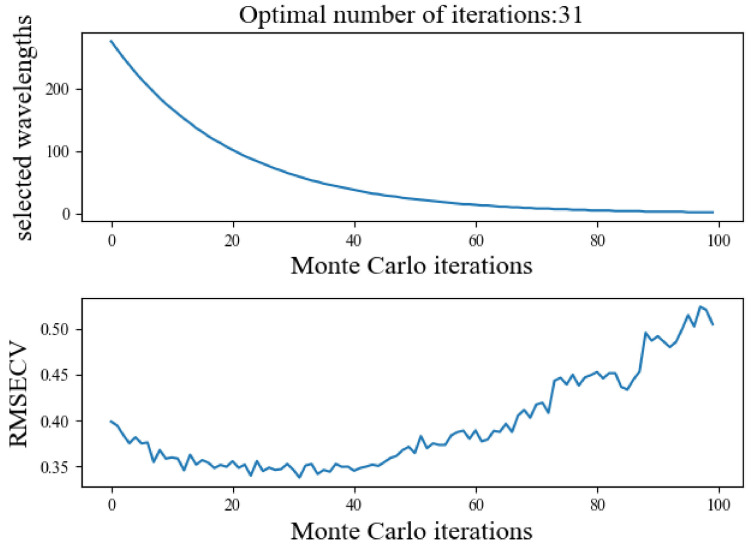
Number of wavelengths and RMSECV in CARS feature wavelength selection.

**Figure 9:**
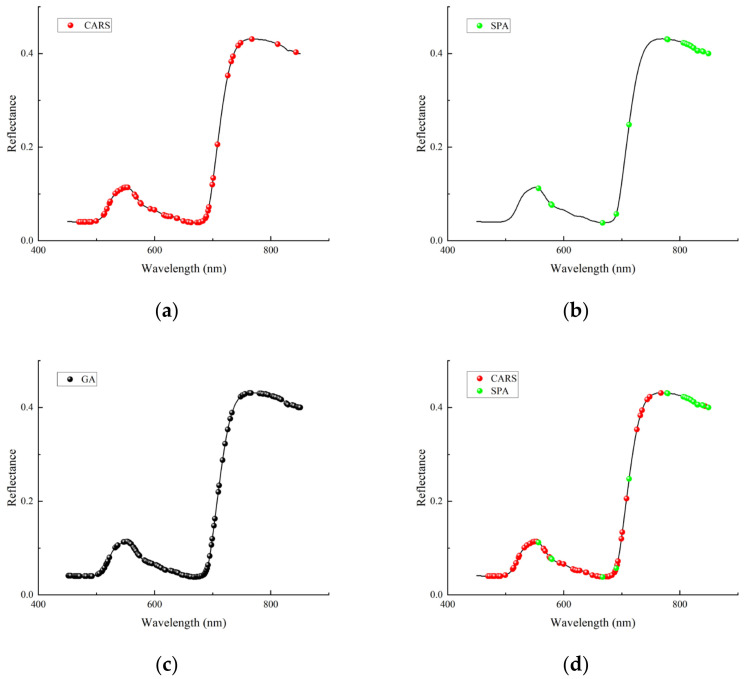
Distribution of feature wavelengths selected by different algorithms: (**a**) CARS; (**b**) SPA; (**c**) GA; (**d**) CARS vs. SPA.

The results of selecting the feature wavelengths using the CARS algorithm are shown in Figure 8. The RMSECV of the model was the minimum when the number of iterations was 31, and 60 feature wavelengths were obtained, whose distribution is shown in Figure 9a. They mainly focused on the 470–700 nm and 725–770 nm regions. The feature wavelengths selected by CARS were numerous and were concentrated in the visible region, except for violet light, with a smaller distribution in the near-infrared region.

The optimal wavelength combination obtained using the SPA algorithm contained 20 wavelengths, whose distribution is shown in Figure 9b, and they were mainly concentrated in the 806–850 nm region. A small number of feature wavelengths were selected by SPA and were distributed in both the visible and near-infrared regions, but wavelengths in the blue region were missing.

There were 127 feature wavelengths selected by the GA algorithm, which is a large number, and their distribution is shown in Figure 9c. They were densely distributed in the visible and near-infrared regions.

As shown in Figure 9d, when comparing the distributions of the two sets of feature wavelengths selected by CARS and SPA over the full band range, it can be found that there is almost no overlap between the selected features, and there is great variability.

#### 3.4.2. Feature Wavelength Selection Based on CARS-SPA-GA

**Figure 10:**
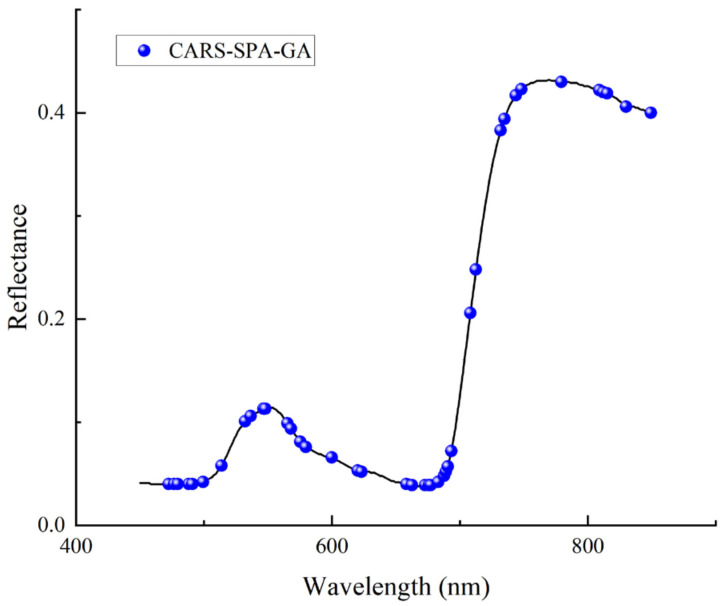
Distribution of feature wavelengths selected by CARS-SPA-GA.

Based on the differences between the features selected by the two algorithms, CARS and SPA, in order to obtain more comprehensive and important features, the experiment combined the two groups of features and removed the duplicates. Then, they were processed to obtain 79 feature wavelengths, which were then subjected to secondary feature selection with the GA. Finally, 40 feature wavelengths were obtained, and their distributions are shown in Figure 10. CARS-SPA-GA selected a more uniform distribution of feature wavelengths in both the visible and near-infrared regions.

#### 3.4.3. Comparison of Results

In order to study the effects of the three feature selection methods used in the experiment to extract the feature wavelengths, as well as the effectiveness of the CARS-SPA-GA method proposed in this paper in extracting the feature wavelengths, we input the full-band data as well as the data of four sets of feature wavelengths into the three models for comparison. We then compared their classification effects under the same model, as well as the overall classification effects, through the evaluation indexes, such as the number of selected bands.

As shown in Table 2, compared with the full-band data, the accuracy and kappa of the output of the bands after feature selection by GA are improved or equalized in all three models, with SVM improving the OA on the test set by more than 1% and reducing the number of filtered bands by half. As a result, it can be seen that there are indeed redundant bands in the hyperspectral data in this paper that interfere with the discrimination of the model. The bands that were feature-selected by CARS showed slight changes in the accuracy and kappa of the outputs in the three models, with SVM and RF decreasing the OA on the test set by about 0.3% and 1%, respectively, and the MLP improving it by about 1%. The SPA-screened bands showed a decrease in OA of less than 3% in all three models. In turn, however, the number of bands screened by CARS was less than 22 percent of the full band, and the number of bands screened by SPA was about 7 percent of the full band. This indicates that all three feature selection algorithms achieved good data dimensionality reduction and did not cause a large loss in accuracy. Especially when the GA was combined with the RCGA-SVM model, the model accuracy was improved, while the data dimension was reduced. Liu Tan et al. [37] also established a hyperspectral disease classification model for rice blast based on SVM, which improved the accuracy of the classification and detection of this disease. In contrast, in the study by Liang Ying et al. [38], the RF model output accuracy of 100%, showing an advantage over machine learning models such as SVM. This indicates that different models are suitable for different types of data and problems.

Since the OA of both sets of feature wavelengths obtained using the two feature selection methods, CARS and SPA, output by the RCGA-SVM model exceeds 95%, it is clear that both sets of bands contain important information. Comparing the feature sets selected by the single CARS method and the SPA method, the number of features selected by the CARS-SPA-GA method was between those selected by these two methods, and the highest OA and kappa were obtained for inputs to the three different types of models. Wavelength selection using only the GA obtained features accounting for about 50% of the full band, whereas the CARS-SPA-GA method used in this experiment, where all feature wavelengths selected by CARS and SPA were selected twice using the GA, selected significantly fewer features, at less than 15% of the full band. Compared to the single GA method, the accuracy decreased by, at most, about 1%, and there was an improvement in the accuracy on the MLP model. Currently, most of the publicly available research on feature selection methods aims to quickly reduce the feature dimensions while ensuring accuracy, such as through correlation analysis and algorithm concatenation. For example, Mei Guangyuan et al. [39] used variable selection in random forests (VSURF) combined with correlation analysis (CA) to screen the feature wavelengths of wheat stripe rust and established a model for its disease index estimation. Wang Yujie et al. [40] coupled variable cluster analysis with an iterative retention algorithm for feature selection to build a terahertz spectroscopy-based identification model for adulterated rice seeds. Different data usually require specific feature selection methods in order to build a more stable model.

**Figure 11:**
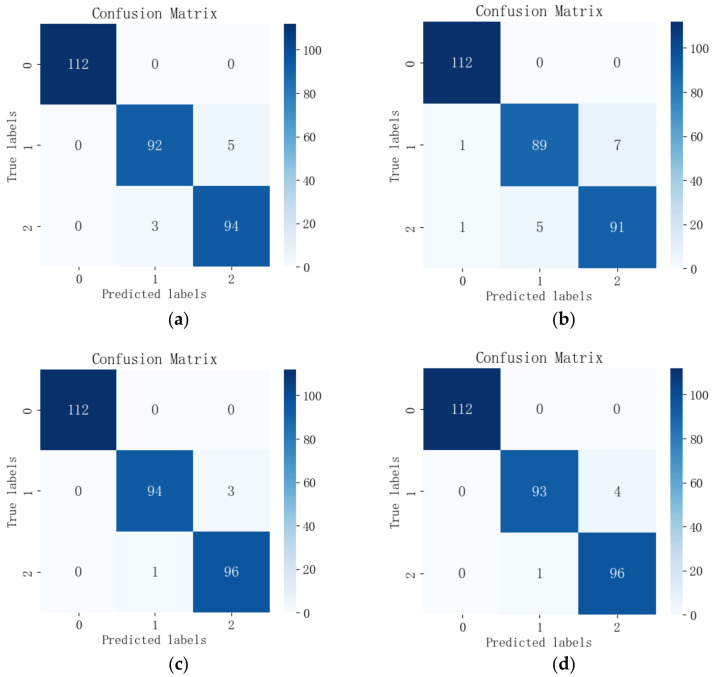
Confusion matrix of feature wavelengths selected by the four algorithms output in the RCGA-SVM model: (**a**) CARS; (**b**) SPA; (**c**) GA; (**d**) CARS-SPA-GA.

**Figure 12:**
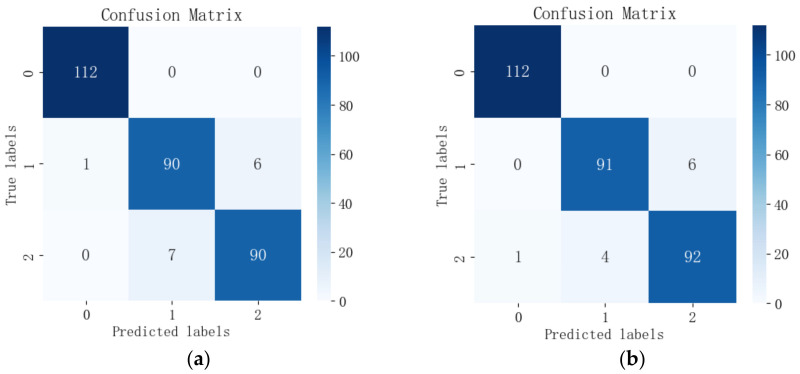

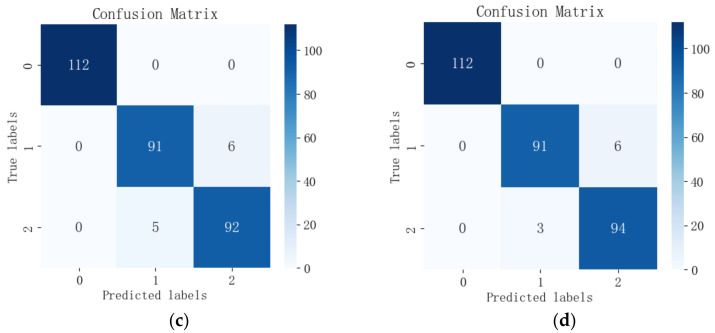
Confusion matrix of feature wavelengths selected by the four algorithms output in the RCGA-RF model: (**a**) CARS; (**b**) SPA; (**c**) GA; (**d**) CARS-SPA-GA.

**Figure 13:**
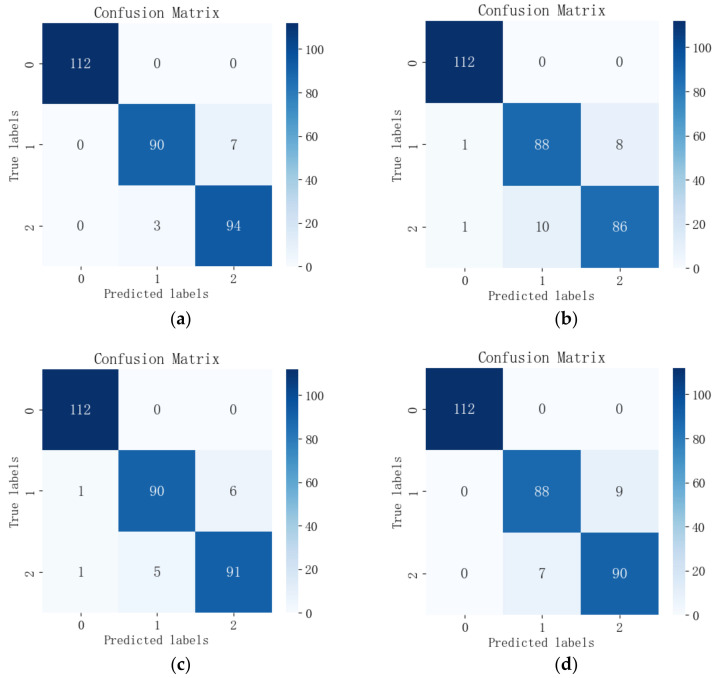
Confusion matrix of feature wavelengths selected by the four algorithms output in the RCGA-MLP model: (**a**) CARS; (**b**) SPA; (**c**) GA; (**d**) CARS-SPA-GA.

The feature wavelengths selected by the four feature selection algorithms were input into each of the three models for comparison, and the output confusion matrices are shown in Figure 11, Figure 12 and Figure 13. Among the 12 combinations of feature wavelengths and classification models, all of them can accurately identify healthy leaves without misclassification. The results showed that the classification difficulties were mainly centered on the distinction between early blight leaves and insect-infested leaves. Combined with Table 2, it can be found that, for the same spectral data, the RCGA-SVM model has 100% accuracy for the classification of the healthy class of data, with individual misclassifications for the two types of pest and disease leaves, and it shows better classification among the three models.

## 4. Conclusions

Taking potato pests and diseases in the fields of Hohhot and Ulanqab in the Inner Mongolia Autonomous Region as the research object, this paper achieved the classification of early blight in potato based on hyperspectral imaging technology, starting from the leaves. Through a large number of experiments, we obtained the CARS-SPA-GA feature selection method and established a potato early blight classification model consisting of RCGA-SVM, reaching the following conclusions.

First of all, compared with the original data, data preprocessing by the MSC method can lead to a substantial improvement in the classification accuracy of the model and achieve better results in different models. Second, in the same model, CARS-SPA-GA selected fewer features and obtained higher accuracy, reducing the data complexity, while the feature wavelengths contained more comprehensive important information. Finally, the RCGA-SVM model significantly outperformed the other two models with the same input bands. Moreover, the model combined with the CARS-SPA-GA feature selection algorithm output accuracy of 98.366%, which is optimal for classification. Therefore, it has been shown that the method proposed in this paper can be used as a reference for related studies, such as those on hyperspectral data classification and feature selection.

Our method performs well in a controlled environment, but we recognize its limitations. The experimental conditions were idealized and the spectral data were measured in a dark box. It should be noted that the lighting conditions are difficult to control in real environments, and this will undoubtedly affect the performance of the model. In future studies, we will collect data and perform additional validation under different environmental conditions to improve the adaptability of the model to environmental changes. We will continue to expand the sample to explore the robustness of the method and models on data from field environments, such as introducing data from potato leaves of similar diseases and data collected in the field environment in situ, which will ensure that the experiment is more practical and informative. We will also learn from the research experience of other scholars in the international arena and study other types of feature selection methods to further improve the model’s performance.

We believe that by addressing these limitations and further optimizing our approach, we can improve its applicability in real-world environments and build a solid foundation for future research and applications.

## Figures and Tables

**Table 1:** Comparison of six preprocessing methods on different classifiers.

Classifier	Method	OA%		Kappa
		Train Set	Test Set	
SVM	-	97.327	96.078	94.103
	MSC	99.015	97.712	96.560
RF	-	95.499	89.542	84.264
	MSC	98.312	96.405	94.594
MLP	-	94.936	94.771	92.137
	MSC	97.468	95.751	93.612

**Table 2:** Comparison of CARS-SPA-GA and classical feature selection methods on different models.

Model	Method	Wavelength	OA%		Kappa
			Train Set	Test Set	
RCGA-SVM	-	276	99.015	97.712	96.560
	GA	127	99.015	98.692	98.034
	CARS	60	98.171	97.385	96.068
	SPA	20	95.499	95.424	93.117
	CARS-SPA-GA	40	98.452	98.366	97.543
RCGA-RF	-	276	98.312	96.405	94.594
	GA	127	98.312	96.405	94.594
	CARS	60	98.030	95.424	93.119
	SPA	20	97.749	96.405	94.593
	CARS-SPA-GA	40	98.030	97.058	95.577
RCGA-MLP	-	276	97.468	95.751	93.612
	GA	127	97.327	95.751	93.609
	CARS	60	96.765	96.732	95.086
	SPA	20	94.092	93.464	90.167
	CARS-SPA-GA	40	97.890	94.771	92.137

## Data Availability

If the data are needed, interested parties may contact the corresponding author.
